# Priming mesenchymal stem cells boosts stem cell therapy to treat myocardial infarction

**DOI:** 10.1111/jcmm.12036

**Published:** 2013-03-14

**Authors:** Juliana L Carvalho, Vinicius B A Braga, Marcos B Melo, Ana Carolina D A Campos, Maira S Oliveira, Dawidson A Gomes, Anderson J Ferreira, Robson A S Santos, Alfredo M Goes

**Affiliations:** a Department of Biochemistry and Immunology, Institute of Biological Sciences, Federal University of Minas GeraisBelo Horizonte, Brazil; b Department of Morphology, Institute of Biological Sciences, Federal University of Minas GeraisBelo Horizonte, Brazil; c Department of Physiology and Biophysics, Institute of Biological Sciences, Federal University of Minas GeraisBelo Horizonte, Brazil; d Department of Clinical and Surgery, College of Veterinary Medicine, Federal University of Minas GeraisBelo Horizonte, Brazil

**Keywords:** adipose tissue stem cells, mesenchymal stem cells, myocardial infarction, cell therapy, connexin-43

## Abstract

Cardiovascular diseases are the number one cause of death globally and are projected to remain the single leading cause of death. Treatment options abounds, although efficacy is limited. Recent studies attribute discrete and ephemeral benefits to adult stem cell therapies, indicating the urge to improve stem cell based–therapy. In this study, we show that priming mesenchymal stem cells (MSC) towards cardiomyogenic lineage enhances their beneficial effects *in vivo* as treatment option for acute phase myocardial infarction. MSC were primed using cardiomyogenic media for 4 days, after which peak expression of key cardiomyogenic genes are reached and protein expression of Cx-43 and sarcomeric α-actinin are observed. MSC and primed MSC (pMSC) were characterized *in vitro* and used to treat infarcted rats immediately after left anterior descending (LAD) occlusion. Echocardiography analysis indicated that MSC-treated myocardium presented discrete improvement in function, but it also showed that pMSC treatment lead to superior beneficial results, compared with undifferentiated MSC. Seven days after cell injection, MSC and pMSC could still be detected in the myocardium. Connexin-43 expression was quantified through immunoblotting, and was superior in pMSC, indicating that this could be a possible explanation for the superior performance of pMSC therapy.

## Introduction

Cardiovascular diseases (CVD) are the number one cause of death globally and are projected to remain the single leading cause of death. CVD are partially the result of unhealthy lifestyle that includes tobacco use, physical inactivity and unhealthy diets. Therefore, they are easily prevented, but not easily treated. The need for increased governmental investment through national programs aimed at research on prevention and treatment of CVD and other noncommunicable diseases is beyond dispute 1.

One of the most important CVD is coronary heart disease, which constitutes a high morbidity life-threatening condition. Coronary heart disease is characterized by occlusion of coronary arteries leading to myocardial infarction, followed by irreversible loss of cardiac cells, ventricular remodelling and finally organ failure, in spite of aggressive pharmacotherapy and surgical procedures. Indeed, spontaneous regeneration of the myocardium has been considered extremely limited for the last several years, and remains to be considered as so, even though a small population of resident cardiac progenitor cells, capable of proliferating and reconstituting the tissue, has been recently described 2.

Treatment of heart failure and heart attack includes oral medication, coronary artery bypass, balloon angioplasty, valve repair and replacement, heart transplantation, and artificial heart surgeries. These strategies are mostly effective, though are also expensive. Furthermore, heart transplantations are hindered by lack of donor hearts, and the other strategies only prevent further infarction events, not restoring the injured myocardium and hence, quality of life. Given the knowledge that a myocardial infarction can result in the loss of over 1 billion cells 3 and that strategies based on preventing cell death are limited, they do not constitute ideal options to actually regenerate and restore myocardial function. To provide myocardium with a source of cells capable of mitosis and regeneration through differentiation into functional myocardium, cell therapy using stem cells is currently being investigated 4, 5, 6.

Present data concerning stem cell effects on myocardial infarction indicates that undifferentiated adult mesenchymal stem cells (MSC) promote benefits to heart function through paracrine signalling, prevention of cell death, diminution of fibrosis, promotion of vascularization and, possibly, through differentiation into cardiomyocytes 7, 8, even though the latter is still under discussion 3, 9, 10. In recent clinical studies, conflicting results are also found. Even though there are studies describing positive and long-term effects of adult stem cells in myocardial function after infarction 11, 12, others account discrete and ephemeral benefits to adult stem cell therapies 13, indicating the urge to improve stem cell based-therapy.

One option to achieve such a goal would be to isolate and differentiate MSC *in vitro* prior to inject them into lesion sites. Currently, though, such strategy is hindered by the fact that MSC differentiation towards cardiomyogenic lineage still remains largely controversial 9, 10. Moreover, completely differentiated cells are more sensitive to the injection procedure, may not synchronize with the tissue and might not survive in high rates 14. In the present work, we showed that it is possible to boost the beneficial effects of MSC therapy by priming cells before injection into infarcted myocardium. Regular and strain echocardiography were used to demonstrate the enhanced myocardial function of primed MSC (pMSC)-treated myocardium during acute phase. Also, we found that higher connexin-43 expression might be involved in the superior results observed in pMSC-treated rats.

## Materials and methods

### Animals

Lewis LEW-Tg (EGFP) F455.5/Rrrc rats 6–8 weeks old, which express enhanced fluorescence green fluorescent protein (EGFP), were obtained from the Rat Resource and Research Center, Missouri, USA. The animals were housed in a climate-controlled environment under a 12-hr light/dark cycle with free access to rat chow and water. All experimental protocols were performed in accordance with the guidelines for the humane use of laboratory animals established at our Institution. This study was approved by the Committee of Ethics in Research at the Federal University of Minas Gerais (Protocol #61/2010).

### Adipose tissue MSC isolation and culture

Adipose tissue–derived MSC were isolated as previously described, with minor modifications 15. Briefly, gonadal adipose tissue depots were collected from 6-week-old rats, washed with phosphate-buffered saline (PBS) and digested with 0.15% collagenase II (Sigma-Aldrich, St. Louis, MO, USA) for 1 hr. Collagenase activity was inhibited by the addition of FBS (Gibco, Grand Island, NY, USA) and the digested tissue was centrifuged at 330 × *g* for 10 min. Pellet was resuspended in basal media and plated in T75 tissue flasks (Techno Plastics Products, SWI). Basal media was composed of 10% FBS in DMEM (Gibco). Cell cultures were kept in a humidified atmosphere with 5% CO_2_ at 37°C for 24 hrs before the first medium change. From the first medium change after, medium was changed every 3 days. The mesenchymal population was isolated based on its ability to adhere on the culture plate. At 80–90% confluence, cells were detached using 0.25% trypsin-EDTA (Gibco) and replated in other flasks at 1:3 ratios. Third passage MSC were used in all experiments 16, 17.

### Flow cytometry analysis

The cell surface antigen profile specific to MSC was characterized by flow cytometry. Briefly, cells were harvested and washed with PBS. Approximately 5 × 10^5^ cells were incubated for 30 min. at 4°C with the following primary antibodies: mouse anti-rat CD45, CD54, CD73 and CD90 (all from Abcam, Cambridge, MA, UK). After washing, cells were incubated with a secondary antibody, the 488-labelled antimouse IgG (Molecular Probes, Eugene, OR, USA), for 30 min. at 4°C, washed again and suspended in PBS. As a control, cells were incubated with only the secondary antibody to exclude nonspecific binding. Quantitative analysis was performed using a FACScan argon laser cytometer (Becton Dickson, San Jose, CA, USA). For each sample, 15,000 events were acquired and analysed with the CELL QUEST software. Cell surface marker expression was determined by comparison with the isotype control on a histogram plot and data analysis was performed with WinMid 2.8 analysis software.

### Cell culture and induction of cardiac program in MSC (MSC Priming)

To induce the cardiac program in MSC (MSC priming), cells were seeded at a density of 6.66 × 10^4^ cells/cm^3^ into T-75 tissue culture plates for 4 days. The induction period of 4 days was established and used in all experiments. The induction medium consisted of DMEM/20% FBS supplemented with recombinant IL-3, IL-6 and SCF, 10^−4^ M of 2-mercaptoethanol, 2 mM of L-glutamine, 200 μg/ml human apo-transferrin (all from Sigma Chemical CO, St Louis, MO, USA) and 10 μg/ml recombinant human insulin (Novolin®), as previously described 18.

### Polymerase chain reaction (PCR)

eGFP gene detection reaction involved genomic DNA isolation using DNAzol reagent and following manufacturer's instructions. A duplex PCR was performed containing the following primers: LWS 455 5F: AACCTCCCAGTGCTTTGAACGCTA, LWS 455 5R: GGTGCAAGCCTCAACTTCTTTGT and U3r-4: ATCAGGGAAGTAGCCTTGTGTGTG. LWS 455 5F anneals with both eGFP^−^ and eGFP^+^ genomic DNA, in contrast to LWS 455 5R, which only anneals with eGFP^−^ and U3r-4, which anneals only to eGFP^+^ genomic DNA. Therefore, the pair of primers LWS 455 5F and LWS 455 5R generate amplicons of 438 bp, and the pair of primers LWS 455 5F and U3r-4 generate amplicons of 128 bp. Homozygous genetic material of eGFP^−/−^ rats/cells/myocardium presents only genomic DNA in which LWS 455 5F and LWS 455 5R anneal, therefore a PCR of such material will lead to the formation of amplicons of 439 bp. On the other hand, genetic material of eGFP^+/+^ rats/cells/myocardium will only anneal with LWS 455 5F and U3r-4, and after PCR will generate 128 bp amplicon. In case of heterozygous or mixed material, which presents cells of different genotypes, both amplicons will be generated, as observed in Figure 4. The amplicon of 128 bp is much less visible than the 439 bp amplicon, due to the fact that it derives from eGFP^+/+^ material, derived from injected MSC (line 3) and pMSC (line 4). Injected cells (eGFP^+/+^) are much more rare in the sample than host tissue cells (eGFP^−/−^).

Reverse transcriptase-polymerase chain reaction was also performed, to assess Nkx2.5, α-MHC and β-MHC expression in pMSC. Total cellular RNA was extracted from MSCs cultured on basal and cardiogenic media for 4 days with Trizol (Invitrogen, Carlsbad, CA, USA), as described by the manufacturer. Total RNA was treated with the RevertAid™H Minus M-MuLV RT (Fermentas, Hanover, MD, USA) to generate cDNA using an oligo(dT) adapter primer. Next, PCR amplification was performed for Nkx2.5, α-MHC, β-MHC and GAPDH. The primers used were Nkx2-5 sense: 5′- CTTCAAGCAACAGCGGTACC-3′, Nkx2-5 antisense: 5′- ATCTTGACCTGCGTGGACG-3′ (25); α-MHC sense: 5′- TGTGAAAAGATTAACCGGAGTTTAA-3′; α-MHC antisense: 5′- TCTGACTTGCGGAGGTATC-3′; β–MHC sense: 5′- AAGTCCTCCCTCAAGCTCCTAAGT-3′, β–MHC antisense: 5′- TTGCTTTGCCTTTGCCC-3′, GAPDH sense 5′- TGCACCACCAACTGCTTA-3′, GAPDH antisense: 5′- GGATGCAGGGATGATGTTC-3′. The PCR cycles were as follows: 94°C for 2 min., 94°C for 30 sec., 60°C for 45 sec. and 72°C for 45 sec. (30 cycles), 72°C for 10 min. The RT-PCR products were analysed through 1% agarose gel electrophoresis and visualized with SYBR Safe DNA Gel Stain (Invitrogen).

### Immunocytochemistry analysis

Cells were washed with PBS and fixed with 4% PFA. Antibodies used to assess cardiomyogenic phenotype identified the following structures: sarcomeric α-actinin and connexin-43 (both from Abcam). Alexa Fluor dye conjugated secondary antibodies (Invitrogen) were used for detection of mouse or rabbit primary antibodies.

### Myocardial infarction (MI) procedures

Under anaesthesia with 10% ketamine-2% xylazine (4:3, 0.1 ml/100 g, i.p.), rats (*n* = 5 in each group) were placed in the supine position on a surgical table, intubated, and ventilated with room air using a respirator for small rodents. The chest was opened by a left thoracotomy at the fourth or fifth intercostal space. To expose the heart, a small-sized retractor was used to maintain the ribs separated. After incision of the pericardium, the heart was quickly removed from the thoracic cavity and turned left to allow access to the proximal left anterior descending (LAD) coronary artery. A 4-0 silk suture was snared around the LAD and tightly ligated to occlude the vessel. The heart was then placed back, and the chest was closed with 4-0 silk sutures 19. Sham-operated rats were treated in the same manner, but the coronary artery was not ligated (sham group). Cell therapy consisted of a single injection of 10^6^ cells *in situ*. In fact, infarcted rats received either saline (infarction and sham groups), 10^6^ undifferentiated MSC (MI + MSC group) or 10^6^ primed MSC (MI + pMSC group) injected in the surround area of the infarcted myocardium. The volume of injections was established as 50–80 μl. The following week after the procedure, the rats received non-anti-inflammatory analgesic (Tramadol hydrochloride – Neoquimica, Anapolis, GO, Brasil). Seven days after induction of infarction, the rats were killed and the presence of MSC/pMSC was assessed through PCR.

### Echocardiography studies

Echocardiographic features were obtained based on the recommendations of the American Society of Echocardiography. All the transthoracic echocardiograms were performed by a single, blinded observer with the use of a Vevo 2100 (Visual Sonics, Toronto, Canada). Bidimensional (2-D), M-mode, Doppler and radial and longitudinal strain examinations were considered. Ventricular function was assessed by M-mode [ejection fraction (EF) and fractional shortening (FS)] and using the Vevo strain software to assess radial strain from bidimensional long axis view of left ventricles (velocity, displacement, strain and strain rate parameters). FS was calculated from the equation FS = (LVd − LVs)/LVd × 100 (%) where LVd is left ventricle diastolic dimension and LVs is left ventricle systolic dimension. The EF was obtained following the Teicholz equation which is EF = (LVVd − LVVs)/LVVd × 100 (%) where LVVd is left ventricle diastolic volume, LVVs is left ventricle systolic volume and LVVd(s) = π L [LVd(s)]^3^/6 where L is ventricular length. The mean values for six M-mode and for six radial strain measures were considered on statistical analysis. Rats (*n* = 5 in each group) were anaesthetized using 2.5% isoflurane for induction and were placed in supine position. Exams were performed before myocardial infarction induction, as well as 1 and 7 days after the surgery.

### Immunoblotting analysis

Briefly, MSC (Control), pMSC (Primed) and heart samples (Heart) – used as positive control – were washed twice with ice-cold PBS, harvested by scraping and lysed in a lysis buffer (150 mM NaCl, 1 mM EDTA, 20 mM Tris-HCL pH 8.0, 0.5% Nonidet P-40). After incubation on ice for 10 min., the cells of each sample were homogenized by vortex and sonicated. The respective homogenates were centrifuged at 16,100 × *g* for 20 min. at 4°C. Protease inhibitors (Sigma-Aldrich) were added to each sample. The protein amount was assessed by the Bradford assay. Thirty micrograms of protein of each group were subjected to SDS-PAGE electrophoresis and after transferred to a PVDF membrane (*n* = 3 in each group). The membrane was blocked with 5% skim milk in TBST (TBS plus 0.1% Tween 20) for 60 min. and then incubated with primary antibody. Commercially available antibodies for Connexin-43 and GAPDH (both form Abcam) were used at 1:5000 and 1:2000 dilutions respectively. GAPDH was used as an input control. Incubations were carried out overnight. After three washes with TBST, the membranes were incubated with peroxidase-conjugated secondary antibody (1:5000) (Sigma-Aldrich) for 1 hr at room temperature. Blots were visualized by enhanced chemiluminescence and quantitatively analysed using ImageJ software.

### Statistical analysis

All echocardiographic variables were tested by normality and performed analysis of variance (anova). Factorial treatment arrangement (4 × 3) was considered, being 4 groups and 3 time-points. Two-way anova was performed followed by Tukey post-test to assess differences among the groups and among the time-points. Significance was considered for 5% (*P* < 0.05). Analysis were performed in R (2.11 version) software program.

## Results

### Isolated cells are MSC

**Fig. 1:**
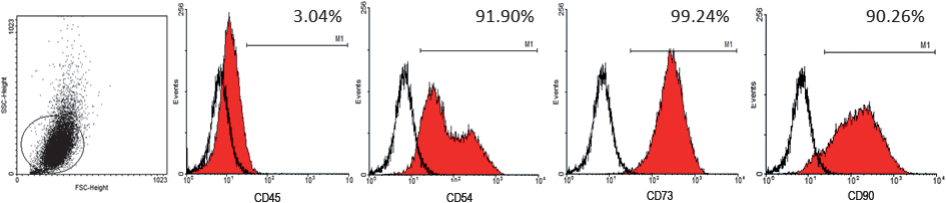
Mesenchymal stem cell characterization. The expression pattern of specific antigens on the surface of the MSC is depicted with representative histograms and the expression of each marker. The cell population expressed CD54, CD73 and CD90, and did not express CD45.

Classification of the cells investigated in the present work was performed as indicated by the International Society for Stem Cell Therapy and the Mesenchymal and Tissue Stem Cell Committee 20. This was performed using both immunostaining (data not shown) and flow cytometry analysis (Fig. 1). As expected, adhesive adipose–derived cells presented the immunophenotype consistent with the accepted definition of MSC, namely: CD45^−^, CD54^+^, CD73^+^ and CD90^+^. Small frequency of CD45^+^ cells suggests that MSC cultures were not contaminated with HSC/progenitor cells. In addition, *in vitro* differentiation using specific differentiation media demonstrated the multipotential property of such cells, able to differentiate into osteoblasts capable of mineralizing ECM and chondrocytes expressing Collagen II mRNA (data not shown).

### Primed MSC (pMSC) express key cardiomyogenic features

**Fig. 2:**
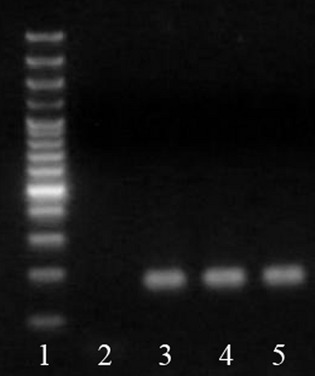
Characterization of MSC primed to follow cardiomyogenic differentiation. pMSC express Nkx2.5, α-MHC and β-MHC mRNA, as depicted. (1) Molecular weight; (2) No DNA control; (3) Nkx2.5 expression; (4) α-MHC expression; (5) β-MHC expression. All samples expressed GAPDH, except no DNA control.

**Fig. 3:**
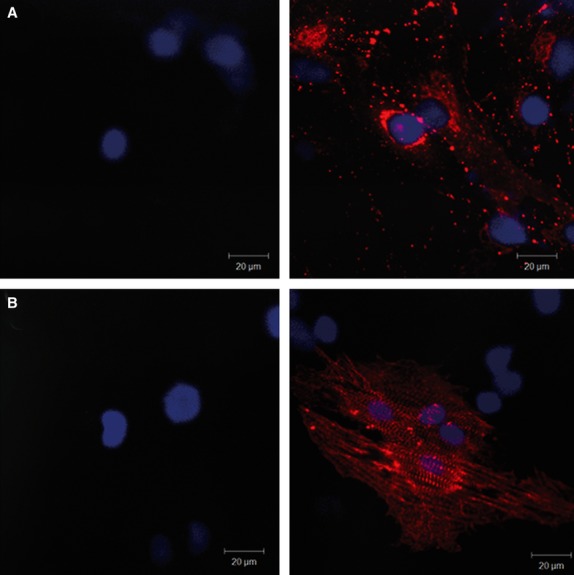
Connexin-43 (**A**) and sarcomeric α-actinin (**B**) protein expression is induced by MSC priming, as depicted in representative confocal images respectively. Pictures in the left column show unprimed MSC and pictures in the right column show pMSC. Only pMSC express connexin-43 and sarcomeric alpha actinin.

To assess MSC priming to the cardiomyogenic lineage, we analysed mRNA expression of key cardiomyogenic genes, indicators of both early and more advanced stages of cardiomyogenic differentiation. After 4 days of culture in cardiomyogenic medium, pMSC started to express Nkx 2-5, as well as α-MHC and β-MHC (Fig. 2), actually reaching peak expression of those genes (Carvalho *et al*., unpublished data). At this time-point, cells also expressed organized sarcomeric α-actinin and connexin-43 (Fig. 3). Furthermore, at 4 days of induction, these cells were not electrophysiologically functional, indicating that they were just primed, but not fully differentiated (data not shown).

### Stem cells are still present at the infarcted myocardium 7 days after cell injection

**Fig. 4:**
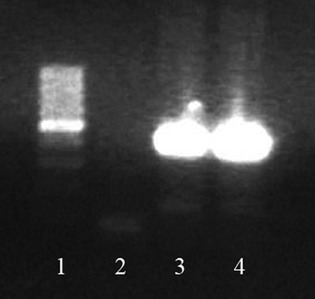
eGFP detection in the heart. Seven days after injection, eGFP^−/−^ myocardium, which received eGFP^+/+^ stem cells presented resident tissue genetic material, but also still presented genetic material from injected cells, indicating their presence in infarcted area. 438 bp amplicon is indicative of eGFP^−^ (wild-type) genetic material, and 129 bp amplicon is indicative of eGFP^+^ (transgenic) genetic material. cDNA produced from control eGFP^−/−^ hearts only present a unique 438 bp amplicon (not shown in the picture). In contrast, eGFP^−/−^ hearts which received eGFP^+/+^ cells not only present a 438 bp amplicon but also a 129 bp amplicon, indicative of eGFP^+/+^ genetic material, derived from injected cells. The 129 bp amplicon is not as common as the eGFP^+/+^ amplicon, due to the fact that injected cells are present in smaller numbers compared with the tissue resident cells. The 129 bp amplicon is still visible in eGFP^−/−^ hearts which received either MSC (line 3) or pMSC (line 4), though. (1) Molecular weight; (2) No DNA control; (3) Infarcted myocardium which received unprimed MSC; (4) Infarcted myocardium which received primed MSC.

Seven days after the myocardial infarction procedure, the presence of GFP^+/+^ cells was assessed through PCR assay. This assay indicated the presence of such cells in infarcted myocardium (Fig. 4). This result indicates that injected cells remain in the damaged area and are still present at the site of injury 7 days after the procedure, acting during the acute phase, as well as the beginning of chronic phase of myocardial infarction.

### pMSC promote major myocardial protection compared with unprimed counterparts

**Fig. 5:**
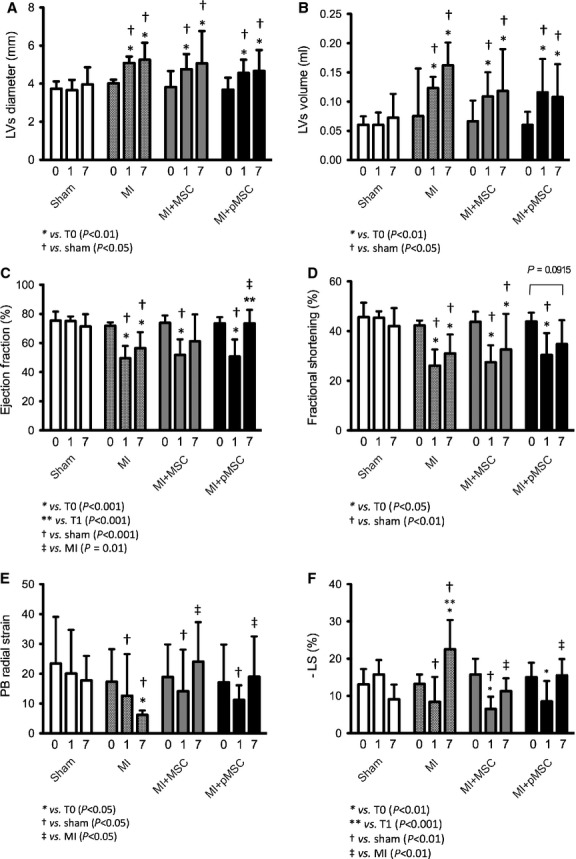
Graphical representation of echocardiographic data. Echocardiographic parameters analysed are represented above, namely: Left ventricle end systolic diameter (**A**) and Volume (**B**), Ejection fraction (**C**), Fractional shortening (**D**), Posterior base radial strain (**E**) and Longitudinal strain (**F**).

**Fig. 6:**
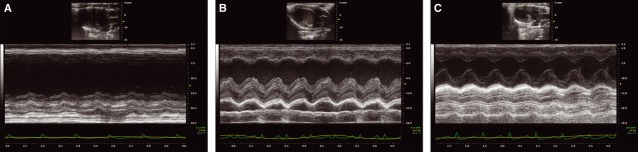
Representative M-mode images showing cardiac function and left ventricle chamber dimensions in MI (**A**), MI + MSC (**B**) and MI + pMSC (**C**) groups.

Echocardiographic results showed that the myocardial infarction was successfully induced in animals and that the stem cell therapy improved cardiac function in those subjects. More specifically, in day 0, prior to intervention, rats from all groups were healthy and had statistically similar cardiac function parameters (data not shown). On day 1 (24 hrs after the intervention), though, only sham group presented similar cardiac function compared with day 0, while the others showed lower (*P* < 0.05) cardiac function, indicating that the myocardial infarction was successfully induced. Results obtained on day 1 indicated significant increase in left ventricle end systolic diameter and volume, as well as decrease in EF, FS, radial velocity, radial displacement, radial strain and longitudinal strain in infarcted rats, irrespective of treatment received (Fig. 5). Finally, on day 7, a cardiac function improvement on animals of the infarction-treated groups was detected, as may be seen in Left Ventricular End Systolic (LVs) Diameter (Fig. 5A) and Volume (Fig. 5B), Ejection Fraction (EF – Fig. 5C), Fractional Shortening (FS – Fig. 5D) and Posterior Base Radial Strain (Fig. 5E) measurements. In addition, echocardiographic data also shows the superior beneficial effects promoted by pMSC compared with MSC treatment. This was observed in M-mode echocardiography EF and FS measurements. According to EF data, MI + pMSC was the only group with significant superior cardiac function in day 7, compared with MI at day 1. Conversely, FS data shows that, at day 7, MI + pMSC was the only group with cardiac function restored to the same levels as day 0 (prior do MI). Those did not happen in MI + MSC group, underscoring that pMSC treatment promoted superior cardiac function improvement compared with MSC treatment. Table 1 summarizes the major echocardiographic findings. Representative of the aforementioned data, M-mode images show the superior contractility of anterior wall of MSC (Fig. 6B) and pMSC (Fig. 6C) treated rats compared with untreated rats (Fig. 6A). In addition to superior anterior wall contractility, pMSC were the only group in which ventricular chamber dilatation was successfully restricted following MI (Fig. 6C).

**Table 1:** Major echocardiography parameters evaluated 7 days after the experimentally induced myocardial infarction

Variables	Groups			
	
	Sham	MI	MI + MSC	MI + pMSC
LVs diameter (mm)	3.96 ± 0.9*	5.25 ± 0.9†,‡	4.67 ± 1.1*,†,‡	5.06 ± 1.7*,†,‡
LVs volume (μL)	72.7 ± 40.6*	162.0 ± 39.1†,‡	118.3 ± 71.6*,†,‡	107.8 ± 56.0*,†,‡
Ejection fraction (%)	71.3 ± 8.5*	56.5 ± 10.9†,‡	61.1 ± 18.9*,†,‡	73.5 ± 9.3*
FS (%)	42.05 ± 7.6*	31.05 ± 7.6†,‡	32.59 ± 14.4†,‡	34.76 ± 9.6*^,†^
LS mean (%)	9.14 ± 4.98*	22.5 ± 17.89^†^	11.3 ± 13.44*^,^‡	15.51 ± 4.4*
PB radial strain (%)	17.74 ± 8.2*	6.20 ± 1.4^†^	24.0 ± 13.2*,†,‡	18.9 ± 13.5*,†,‡

MI: myocardial infarction; MI + MSC: myocardial infarction treated with undifferentiated mesenchymal stem cells; MI + pMSC: myocardial infarction treated with primed mesenchymal stem cells; LVs: left ventricle end systolic; FS: Fractional shortening; LS: Longitudinal Strain; PB: Posterior Base.

*,^†^ Differences between the groups at day 7 (*P* < 0.05).

‡ Different from day 0 inside the group (*P* < 0.05).

### Superior beneficial effects of pMSC on myocardial function may be due to higher connexin-43 expression

**Fig. 7:**
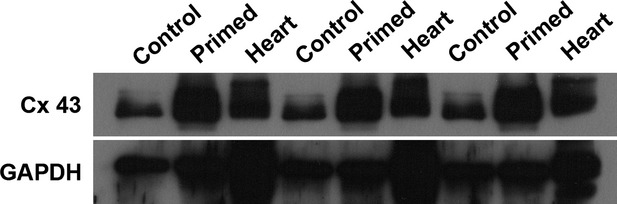
Connexin-43 quantification through immunoblotting. The figure presents the triplicate experiment of Connexin-43 detection and quantification, performed through immunoblotting and normalization to ubiquitous gene GAPDH. In contrast to undifferentiated/unprimed MSC (Control), primed MSC (Primed) express higher levels of Connexin-43. As a positive control, Heart samples (Heart) were also collected and shown. In summary, Connexin-43 expression increases after MSC priming, as shown.

To investigate a possible mechanism underlying the superior performance of primed stem cells on the tissue function preservation, we performed a Western blot assay to quantify the connexin-43 expression in the cells. Connexin-43 constitutes an important protein to be analysed in any study discussing cell therapy for the myocardium, amassing several data indicating its importance for cell–cell interaction, as well as synchronization in the tissue 21. Here, we were able to show that primed stem cells express higher levels of connexin-43 compared with unprimed stem cells, which is another indicative of superior effect of pMSC in repairing infarcted myocardium (Fig. 7).

## Discussion

In the present work, we demonstrated the beneficial effects of priming MSC prior to injection *in situ* to treat myocardial infarction, and assessed a possible mechanism for the observed protective effects of treatments tested. As described in the literature, *in situ* injection is effective in maintaining cells at the place of injury, in opposition to systemic delivery of the cells, hindered by cell entrapment in the lungs and other organs 22. It has also been described that early stem cell treatment of myocardial infarction promotes preservation of tissue function, even though it only promotes limited and ephemeral beneficial effects 13. Therefore, stem cell therapy requires improvement. Here, we described that priming MSC before injecting them *in situ* constitutes an effective, as well as rapid strategy to improve stem cell performance.

Priming stem cells is not such a novelty in the field, and it has been already tested and proved effective in other models, such as the model of immune modulation 23. Actually, priming MSC with Interferon boosts MSC immunosuppressive properties. On the other hand, priming MSC also has the potential to convert immunosuppressive MSC into immuno activating counterparts 24. In this study, we primed MSC to direct them to follow the cardiomyogenic differentiation. As pMSC were not yet electrophysiologically functional after priming, nor completely differentiated, we evaluated a possible mechanism to explain the beneficial effects of priming MSC towards the cardiomyogenic lineage.

In accordance to previous data 13, injection of stem cells immediately after myocardial infarction promoted better heart function. On the other hand, injection of pMSC promoted superior beneficial effects, as indicated in the echocardiographic analysis (Fig. 5 and Table 1). Echocardiography evaluation showed consistent superior myocardial contraction capacity, as reflected by the better LVs Diameter and Volume, EF, FS and PBRS measurements of infarction-treated animals when compared with non-treated infarcted rats at day 7, as well as to intragroup comparison with day 0 (prior to MI). Furthermore, echocardiography data also underscored the superior beneficial effects of pMSC over MSC treatment. Actually, EF data showed that only MI + MSC group had statistically similar ejection fraction as non-treated infarction on day 7, while MI + MSC group did not promote such heart function improvement. Conversely, FS measurements indicated that only MI + pMSC group restored heart function to the levels observed prior to MI at day 7.

The primming method used in this study is based on a protocol previously published by Planat-Benard, in which adipose tissue–derived MSC give rise to functional cardiomyocytes after 28 days of culture in specific media. In this study, we only primed adipose tissue–derived MSCs, maintaining them in the same media as used by Planat-Benard for a short period of 4 days. It was not our intention to obtain beating cardiomyocytes for cell therapy, as fully differentiated cells may be more sensitive to trypsinization and injection protocols. Even though not completely differentiated, pMSC presented characteristics compatible and indicative of cardiac differentiation. Sarcomeric α-actinin expression is an indicative of muscular differentiation, and in association with connexin-43 GAP-junction protein, indicates consistent priming towards cardiomyogenic phenotype, as skeletal myocytes do not present connexin-43. The analysis of such proteins was performed due to their paramount roles in determining MSC cardiomyogenic differentiation, as well as the potential to synchronize with the tissue. Previous clinical trials have indicated the importance of connexin-43 expression for cardiac cell therapy, as observed by the MAYOHEART cell randomized trial, in which connexin-43 negative skeletal muscle myocytes were applied for stem cell therapy, but ended in one death and six patients experiencing treatment related to cardiac arrhythmias 25.

Besides being important for synchronizing with the contracting tissue, connexin-43 has also been described as an essential part of the MSC tools to promote tissue preservation following damage 26. It has been shown that in addition to secrete anti-inflammatory molecules, MSC also interact directly with resident cells of the tissue. Connexin-43 is paramount for MSC to pass survival factors to resident cells, enhancing their resistance to adverse situations such as hypoxia, therefore diminishing apoptosis and enhancing tissue function through tissue preservation 27. Indeed, blocking GAP-junctions actually disturbs MSC-mediated cell protection. Conversely, enhancing connexin-43 expression contributes to enhanced MSC survival following injection 26, as well as to major cell incorporation into the tissue. Finally, it can also lead to a major interaction of pMSC and resident cardiomyocytes. Taken together, the effects of enhancing connexin-43 expression enhances the beneficial effects of MSC cell therapy, leading to major MSC-mediated tissue preservation effect, as already observed in other cells such as astrocytes, where gap junctions composed of connexin-43 reduced apoptotic neuronal damage in cerebral ischaemia 28. Therefore, a higher expression of such protein could be involved in the superior results observed after pMSC treatment.

In summary, marginal results presented by adult stem cell trials treating myocardial infarction have been associated with limited plasticity of adult stem cells. Adult stem cells are still considered an interesting option of cell therapy, though, due to limitations of other options for cell therapy, namely: potentially tumour generating embryonic stem cells, or rare and hardly isolated cardiac stem cells 29. Therefore, boosting adult stem cell efficacy in treating myocardial injuries may be the ideal solution to provide patients with effective, easily isolated, safe non-tumour forming cells. In this study, we have successfully achieved such goal, boosting MSC therapy through priming towards cardiomyogenic lineage.
